# A High Resolution Computer Tomography Scoring System to Predict Culture-Positive Pulmonary Tuberculosis in the Emergency Department

**DOI:** 10.1371/journal.pone.0093847

**Published:** 2014-04-11

**Authors:** Jun -Jun Yeh, Choo-Aun Neoh, Cheng-Ren Chen, Christine Yi-Ting Chou, Ming-Ting Wu

**Affiliations:** 1 Ditmanson Medical Foundation Chia-Yi Christian Hospital, Chiayi, Taiwan; 2 Chia Nan University of Pharmacy and Science, Tainan, Taiwan; 3 Meiho University, Pingtung, Taiwan; 4 Pingtung Christian Hospital, Pingtung, Taiwan; 5 National Yang-Ming University, Taipei, Taiwan; 6 Section of Thoracic and Circulation Imaging, Department of Radiology, Kaohsiung Veterans General Hospital, Kaohsiung, Taiwan; Institute of Infectious Diseases and Molecular Medicine, South Africa

## Abstract

This study evaluated the use of high-resolution computed tomography (HRCT) to predict the presence of culture-positive pulmonary tuberculosis (PTB) in adult patients with pulmonary lesions in the emergency department (ED). The study included a derivation phase and validation phase with a total of 8,245 patients with pulmonary disease. There were 132 patients with culture-positive PTB in the derivation phase and 147 patients with culture-positive PTB in the validation phase. Imaging evaluation of pulmonary lesions included morphology and segmental distribution. The post-test probability ratios between both phases in three prevalence areas were analyzed. In the derivation phase, a multivariate analysis model identified cavitation, consolidation, and clusters/nodules in right or left upper lobe (except anterior segment) and consolidation of the superior segment of the right or left lower lobe as independent positive factors for culture-positive PTB, while consolidation of the right or left lower lobe (except superior segment) were independent negative factors. An ideal cutoff point based on the receiver operating characteristic (ROC) curve analysis was obtained at a score of 1. The sensitivity, specificity, positivity predictive value, and negative predictive value from derivation phase were 98.5% (130/132), 99.7% (3997/4008), 92.2% (130/141), and 99.9% (3997/3999). Based on the predicted positive likelihood ratio value of 328.33 in derivation phase, the post-test probability was observed to be 91.5% in the derivation phase, 92.5% in the validation phase, 94.5% in a high TB prevalence area, 91.0% in a moderate prevalence area, and 76.8% in moderate-to-low prevalence area. Our model using HRCT, which is feasible to perform in the ED, can promptly diagnose culture-positive PTB in moderate and moderate-to-low prevalence areas.

## Introduction

Tuberculosis (TB) outbreaks are common in hospitals, and delayed diagnosis of hospitalized patients with active pulmonary tuberculosis (PTB) is an important factor in nosocomial infections [1]. Many patients experience delays in diagnosis, which can be due to varied symptoms and atypical chest X-ray (CXR) findings [2], [3]. Proposed models to predict culture-positive PTB are based on medical history, clinical symptoms and signs, and chest radiographs [2], [4]–[6]. However, testing ability with respect to post-test probability was reported in only one study [5].

Chest computed tomography (CT), particularly high-resolution computed tomography (HRCT), is feasible to perform in the emergency department (ED), and is well-suited to reveal changes in lung structure [7]–[9]. It has been shown that HRCT can detect culture-positive PTB and predict the risk of sputum smear-negative and sputum-positive PTB [8]–[10]. A recent study has reported the cost-effectiveness of using HRCT for detecting culture-positive PTB [10].

The goal of this study is to investigate the efficacy of a HRCT screening protocol for detecting the presence or absence of culture-positive PTB, and to examine the post-test probability in areas with different prevalence of tuberculosis [5], [11]–[14].

## Materials and Methods

### Study Design

This study was approved by the Ethics Review Board of Ditmanson Medical Foundation Chia-Yi Christian Hospital. As the derivation phase was a retrospective review of medical records, the requirement of informed patients consent was waived. All participants in validation phase of this study signed an informed consent document after being fully informed of the study protocol.

**Figure 1:**
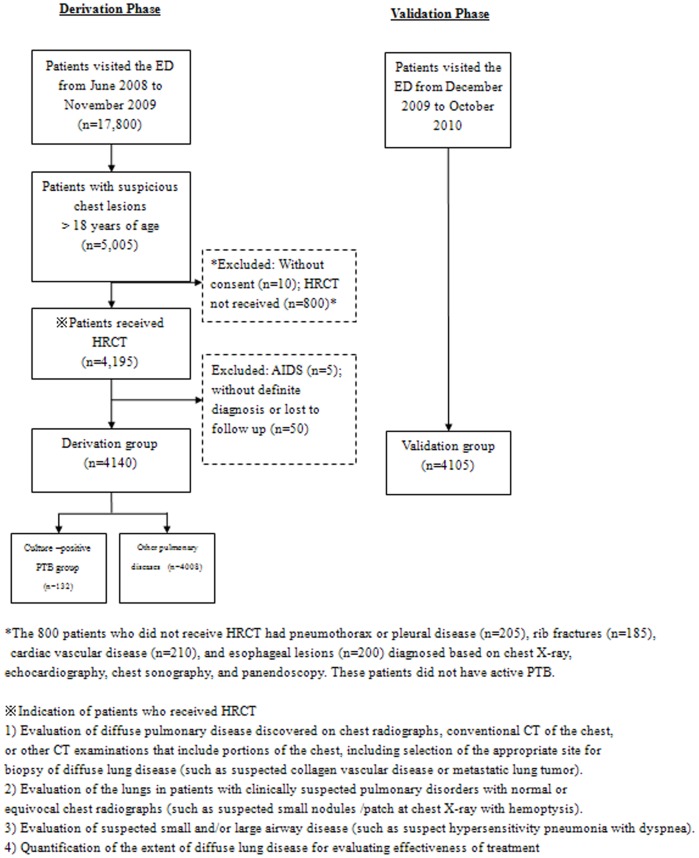
Flowchart of study design.

This was a two-phase study that first identified risk factors for culture-positive PTB in Southern Taiwanese, and then validated those factors. The patients in this study were divided into two groups, those with culture-positive PTB and those with other pulmonary diseases. The overall study design is illustrated in the flowchart presented in Figure 1.

### Derivation Phase

The indications for the use of HRCT of the lungs included the following [15]–[17]. Evaluation of diffuse pulmonary disease discovered on chest radiographs, conventional CT of the chest, or other CT examinations that include portions of the chest, including selection of the appropriate site for biopsy of diffuse lung disease. 2) Evaluation of the lungs in patients with clinically suspected pulmonary disorders with normal or equivocal chest radiographs. 3) Evaluation of suspected small and/or large airway disease. 4) Quantification of the extent of diffuse lung disease for evaluating effectiveness of treatment. There were no absolute contraindications for HRCT of the lungs. Patients with lesions such as pneumothorax (indicated by CXR), rib fractures (indicated by CXR), mediastinal disease, cardiovascular diseases (diagnosed by echocardiography), esophageal lesions (diagnosed by panendoscopy), pleural effusion (diagnosed by chest sonography), and those <18 years of age (to reduce radiation exposure) were excluded from receiving HRCT imaging. Heitkamp et al. [18] and Kirsch et al. [19] published reports after our study which agree with the inclusion and exclusion criteria used in this study.

A total of 15,800 patients visited the ED of our hospital from June 2008 to November 2009. The records of 5,005 patients who were older than 18 years with suspicious pulmonary lesions seen in the ED were retrospectively reviewed [15], [20], [21]. The diagnosis of culture-positive PTB, inactive PTB [22], and non-tuberculosis mycobacterium (NTM) infection [23] were based on culture; diagnosis of pneumonia was based on previous study [24]. The diagnoses of chronic pulmonary diseases were based on pulmonary function tests (PFTs) and clinical history, diagnosis of congestive heart failure was based on echocardiography and clinical history, diagnosis of collagen vascular disease was based on serum titers and pathology, and diagnosis of lung cancer, lymphoma, or metastatic cancer was based on pathology and clinical history [25].

Of the 5,005 patients, 4,195 received HRCT imaging. The 800 patients who did not receive HRCT had a minimal pneumothorax or pleural disease (n = 205), rib fractures (n = 185), cardiac vascular disease (n = 210), and esophageal lesions (n = 200) diagnosed based on chest x-ray, echocardiography, chest sonography, and panendoscopy. These patients did not have culture-positive PTB. In addition, consent had not been received from 10 patients, five patients with AIDS were excluded, and 50 patients did not have a definite diagnosis or were lost to follow-up. Thus, the derivation groups consisted of a total of 4,140 patients.

### Validation Phase

Guidelines were developed from the identified HRCT factors (as explained in the subsequent section) to guide choices regarding in-hospital isolation of patients with culture-positive PTB who were admitted from the ED. This validation phase prospectively validated these guidelines by evaluating their ability to diagnose 4,105 adult patients with suspicious pulmonary lesions admitted from the ED between December 2009 and October 2010. These patients were enrolled with the same inclusion and exclusion criteria as the patients in the derivation phase.

### HRCT Imaging

All patients received chest CT scans with a 64-MDCT scanner (Brilliance, Philips Medical Systems, Cleveland, OH, USA) set to 0.625 mm collimation, 100–120 kV, 250 mAs, a table speed of 57.5 mm/sec, a rotation time of 0.75 sec, and a pitch of 1.07. The images were acquired during a single breath-hold lasting 5–8 seconds, which rendered respiratory motion artifacts uncommon. The spiral mode was used to scan the whole thorax, and the total radiation dose was about 7.0 mSv. The raw data were 0.625 mm (conventional CT is 5 mm thick), and CT reformation yielded HRCT images that were 1 mm thick. The images were reconstructed with a 1-mm slice thickness in the axial plane (no gap) and in the coronal plane (5-mm apart) using a high spatial-frequency algorithm, and then sent to the picture archiving and communication system (PACS) for review. All thin-section multidetector CT (MDCT) images were displayed on a monitor at the pulmonary window level setting (level, -600 HU; width, 1200 HU).

### HRCT Evaluation

#### CT Morphology and anatomy distribution

Definitions of morphology and anatomical distribution were adopted from previously described information [9], [26], [27].

#### Image Interpretation Criteria

The HRCT scans were evaluated by 3 radiologists. Each had over 15 years of experience reading thoracic radiological studies, and was unaware of the sputum smear and clinical examination results. All patients in the study received a chest x-ray, and the x-ray results were available to the radiologists. The request form for the CT examination did not provide any clinical details or any suggestions as to the possible clinical diagnosis. All three radiologists thoroughly read and interpreted all CT images independently on a daily basis. The radiologists thoroughly interpreted the CT images including all 18 segments over both lungs without any focus or view of interest. The locations of lung involvement were reported as one or more of the 18 designated lung segments. In the late afternoon every day, all three radiologists discussed any discrepancies in their findings and any were resolved by consensus.

#### Development of Derivation Set and Validation of Receiver Operating Characteristic (ROC) Curve

**Figure 2:**
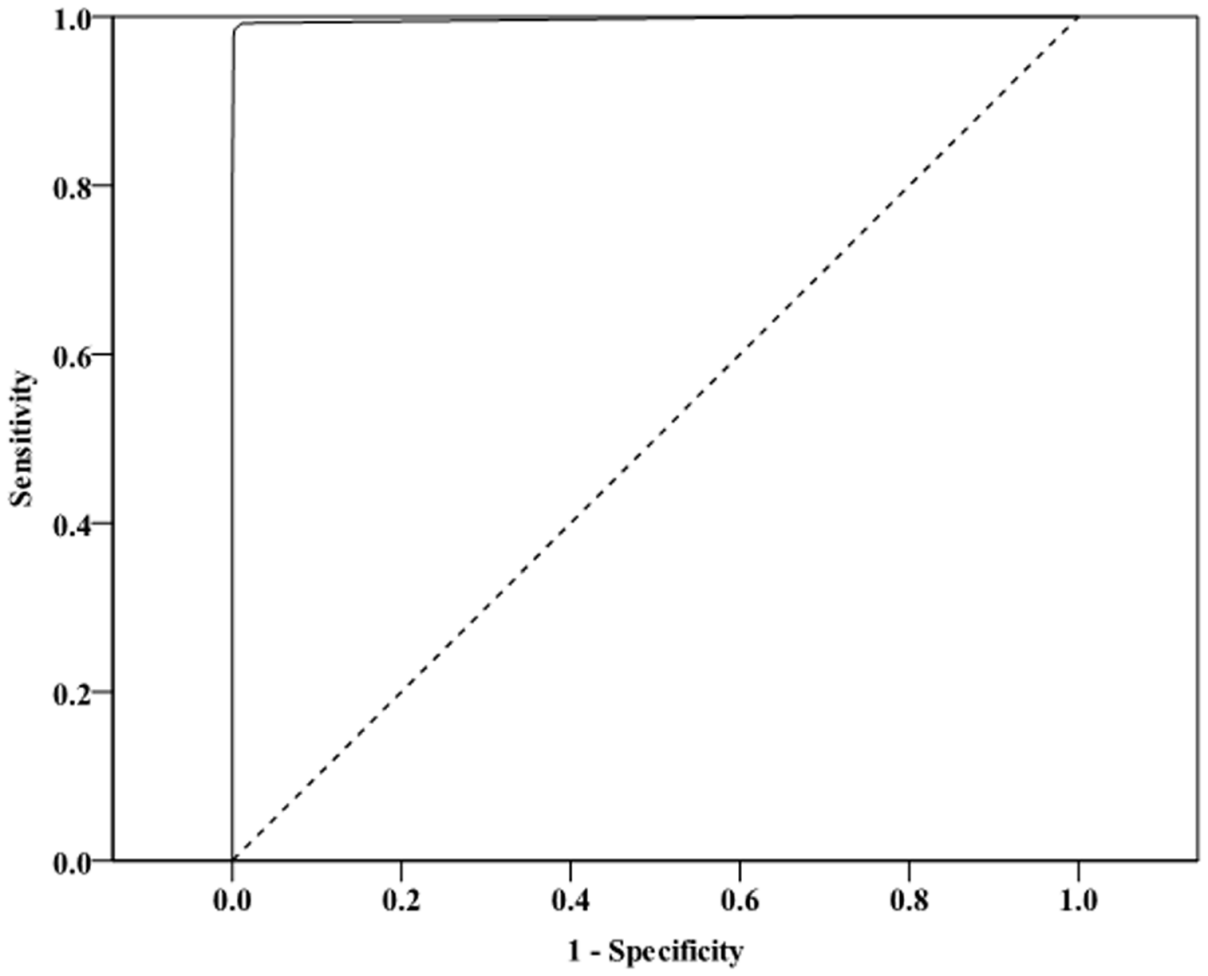
Receiver operating characteristic (ROC) curve from the multivariate logistic regression model analysis of the derivation group. The area under the ROC (AUC) = 0.997 (95% confidence interval [CI] 0.991 to 1.000, *P*<0.001).

We identified predictors of culture-positive PTB in a stepwise logistic regression analysis by considering CT findings that included CT morphology, anatomic distribution, and number of areas of consolidation, cavitations, and clusters of nodules. We identified potential predictive variables for culture-positive PTB using univariate analysis, in which variables with *P*≤0.1 were entered into the multivariate models [5]. Then we used a backward elimination process and maintained variables with *P*<0.001 to derive an index based on a scoring system [28]. The scoring system weighted each variable based on the β-coefficient from the logistic regression analysis. Analysis of the ROC curve found an advantage to using logistic regression weights, so those were used for the scores predicting culture-positive PTB based on the first phase of the study (Fig. 2). We calculated the culture-positive PTB score for each subject by summing the component variables, and we determined a cutoff value (C value) from the prediction model. The second phase of the study validated the ability of the model to predict culture-positive PTB.

#### Care Protocol and Measurements

During the validation phase, we reviewed chest radiographs and HRCT scans of patients, along with their charts for previous PTB, diabetes mellitus, steroid usage, gastrectomy, anemia, and liver cirrhosis. For all cases in the culture-positive PTB groups, we determined if the emergency physician had ordered respiratory isolation and if the diagnosis was confirmed by the results of sputum or other specimens after invasive procedure such as bronchoscopy, pleural biopsy, or surgical intervention. We did not use a standardized guideline for respiratory isolation upon admission during the first phase of the study. During the second phase of the study, patients were admitted to a respiratory isolation setting if their score was over 1 based on the ROC curve. In the validation phase, if the score was >1 upon reading the HRCT, the radiologist notified the attending physician and patient was placed in respiratory isolation.

Post-test probability was subsequently calculated according to the given prevalence and predicted positive likelihood ratio (LR).

### Statistical Analysis

Statistical analyses were performed with SPSS 15.0 statistics software (SPSS Inc., Chicago, IL, USA). Continuous data were presented as mean ± standard deviation, and categorical data by group as number with percentage (%). Two-sample t-test was performed to compare the differences between groups for continuous data. The Pearson's chi-square test or Fisher's exact test was used to compare differences in categorical data between groups. A multiple logistic regression model was performed to identify the predictors of culture-positive PTB. The estimated beta (β) with standard error (SE) and odds ratio (OR) with 95% confidence interval (CI) were calculated for the multivariate logistic regression. A relative score was given by using the lowest β value as a base (here, the β value of cavitation of s1, s2, and s1+s2 was the lowest). For the other variables selected in multivariate logistic regression, the relative score was given as 2 when the ratio (β/5.060) was >1 and <1.5, and as 3 when the ratio was ≥1.5 and <2.5. Since the effect of consolidation of s7, s8, s7+s8, s9, s10 was inverse (β value is negative), the relative score was set as negative. The area under the ROC curve (AUC) indicated the best cutoff point based on maximization of the Younden index. All statistical analyses were considered significant at *P*<0.05.

## Results

The model was derived from 4,140 patients (2698 males), and validated with 4,105 patients (2,684 males). The age and sex distributions of the derivation group and validation group showed no significant difference (both, *P*>0.05; results not shown). Among the patients in the derivation phase, 132 patients (87 male/45 female) were diagnosed with culture-positive PTB, while the others (2611 male/1397 female) were considered to have other pulmonary diseases. Demographic characteristics, medical history, and clinical symptoms and signs were all similar between the culture-positive PTB and the other pulmonary diseases groups. The frequency of smear-positive, culture-positive and smear-negative, culture-positive PTB were different between the derivation phase and the validation phase, but the frequency of culture-positive PTB was similar between the two phases (3.2% [132/4140] vs. 3.6% [147/4105], P = 0.654) (Table 1).

**Table 1:** Demographic and clinical characteristics of the subjects in derivation phase and validation phase.

	Derivation Phase (n = 4,140)			Validation Phase (n = 4,105)		
Variables	G1 (n = 132)§	G2 (n = 4008)	*P*	G3 (n = 147)§	G4 (n = 3958)	*P*
Age, y	66.6±10.8	67.1±9.2	0.573	67.7±10.2	66.9.1±8.2	0.471
Sex, males	87 (65.9)	2611 (65.1)	0.856	98 (66.7)	2586 (65.3)	0.739
Anemia (<11 g/dL)	51 (38.6)	1338 (33.4)	0.209	53 (36.1)	1305 (33.0)	0.435
Gastrectomy	15 (11.4)	357 (8.9)	0.332	16 (10.9)	355 (9.0)	0.426
Diabetes mellitus	63 (47.7)	1710 (42.7)	0.247	67 (45.6)	1691 (42.7)	0.492
Alcoholism	14 (10.6)	318 (7.9)	0.266	15 (10.2)	315 (8.0)	0.344
Received steroids	44 (33.3)	1077 (26.9)	0.100	49 (33.3)	1060 (26.8)	0.079
Albumin <2.5 g/dL	52 (39.4)	1327 (33.1)	0.132	46 (31.3)	1312 (33.1)	0.639
Smear-positive, culture positive	108 (81.8)	0 (0)	<0.001*	100 (68.0)	0 (0)	<0.001*
Smear-negative, culture positive	24 (18.2)	0 (0)	<0.001*	47 (32.0)	0 (0)	<0.001*
Bacterial infection (blood culture/effusion/sputum)	0 (0)	2786 (69.5)	<0.001*	1 (0.7)*	2829 (71.5)	<0.001*
Mycoplasma infection (elevated titer)	0 (0)	261 (6.5)	<0.001*	0 (0)	256 (6.5)	<0.001*
Viral infection (elevated titer or pathology)	0 (0)	1 (0)	1.000	0 (0)	0 (0)	NA
Non-tuberculosis mycobacterial infection (culture)	0 (0)	136 (3.4)	0.022	0 (0)	101 (2.5)	<0.001*
Fungus (pathology)	0 (0)	4 (0.1)	1.000	0 (0)	3 (0.1)	1.000
Congestive heart failure	0 (0)	25 (0.6)	1.000	0 (0)	19 (0.5)	1.000
Chronic bronchitis	0 (0)	325 (8.1)	<0.001*	0 (0)	337 (8.5)	<0.001*
Collagen vascular disease	0 (0)	20 (0.5)	1.000	0 (0)	17 (0.4)	1.000
Lung cancer/lymphoma/metastatic cancer to lung (pathology)	0 (0)	450 (11.3)	<0.001*	1 (0.7)*	396 (10.0)	<0.001*
**Symptoms and signs**						
Fever	53 (40.2)	1342 (33.5)	0.111	60 (40.7)	1858 (46.9)	0.144
Weight loss	51 (38.6)	1339 (33.4)	0.211	59 (40.1)	1619 (40.9)	0.914
Cough	50 (37.8)	1338 (33.4)	0.337	87 (59.2)	2601 (65.7)	0.112
Weakness	56 (42.4)	1579 (39.4)	0.484	61 (41.5)	1616 (40.8)	0.933

G1, patients with culture-positive PTB in derivation group; G2, patients other pulmonary diseases in the derivation group; G3, patients with culture-positive PTB in validation group; G4, patients other pulmonary diseases in the validation group.

Data are presented as mean ± standard deviation for continuous variables, and n (%) for categorical variables.

PTB, pulmonary tuberculosis; NA, not assessed.

*Indicates statistical significance between G1 and G2 in derivation phase or between G3 and G4 in validation phase, *P*<0.05.

§ Comparision of the incidence of culture-positive PTB of the derivation group with the validation group (3.2% [132/4140] vs. 3.6% [147/4105], P = 0.654).

Note: Combined disease such as bacterial infection with culture-positive PTB (n = 1), and lymphoma with culture-positive PTB (n = 1) were grouped as culture-positive PTB. In the derivation phase there were 633 patients with previous PTB (19 with culture-positive PTB and 614 other pulmonary diseases without culture-positive PTB).

When CT morphology and anatomic examinations were compared between the culture-positive PTB and other pulmonary diseases groups, the culture-positive PTB group had higher values in consolidation, cavitation, clusters of nodules, ground-glass-opacity, and centrilobular nodules with tree-in-bud appearance, but had lower values in fibrosis (all, *P*<0.05) (Table 2). Anatomic examination found that the culture-positive PTB group had significantly higher values for consolidation of s1, s2, s1+s2, s3, s4, s5, and s6, for cavitation of s1, s2, s1+s2, s3, s4, s5, and s6, and for all the clusters of nodules/mass. Vice versa, lower values for consolidation of s7, s8, s7+s8, s9, s10 were found in the culture-positive PTB group (all, P<0.05). The kappa value for both inter-observer and intra-observer variation (including the interpretation of HRCT morphology and the score of HRCT report) was >0.9 indicating excellent reliability.

**Table 2:** High-resolution computed tomography findings of subjects in the derivation phase and validation phase.

	Derivation Phase (n = 4,140)			Validation Phase (n = 4,105)		
Variables	G1 (n = 132)	G2 (n = 4,008)	*P*	G3 (n = 147)	G4 (n = 3,958)	*P*
**Consolidation**						
s1, s2, s1+s2	102 (77.3)	305 (7.6)	<0.001*	104 (70.7)	317 (8.0)	<0.001*
s3, s4, s5	20 (15.2)	289 (7.2)	0.002*	15 (10.2)	296 (7.5)	0.220
s6	66 (50)	65 (1.6)	<0.001*	70 (47.6)	64 (1.6)	<0.001*
s7, s8, s7+8, s9, s10	11 (8.3)	1356 (33.8)	<0.001*	13 (8.8)	1342 (33.9)	<0.001*
**Cavitation**						
s1, s2, s1+s2	81 (61.4)	34 (0.8)	<0.001*	72 (49.0)	10 (0.3)	<0.001*
s3, s4, s5	8 (6.1)	88 (2.2)	0.004*	7 (4.8)	64 (1.6)	0.004*
s6	36 (27.3)	98 (2.4)	<0.001*	32 (21.8)	72 (1.8)	<0.001*
s7, s8, s7+8, s9, s10	8 (6.1)	88 (2.2)	0.004*	5 (3.4)	149 (3.8)	0.820
**Clusters of nodules**						
s1, s2, s1+s2	102 (77.3)	6 (0.1)	<0.001*	97 (66.0)	0 (0)	<0.001*
s3, s4, s5	17 (12.9)	2 (0.05)	<0.001*	11 (7.5)	0 (0)	<0.001*
s6	26 (19.7)	4 (0.1)	<0.001*	23 (15.6)	3 (0.1)	<0.001*
s7, s8, s7+8, s9, s10	23 (17.4)	2 (0.05)	<0.001*	22 (14.9)	0 (0)	<0.001*
**Interlobular septal thickening**	93 (70.5)	2231 (55.7)	0.001*	89 (60.5)	2215 (56.0)	0.272
**Bronchial wall thickening**	103 (78.0)	2647 (66.0)	0.004*	106 (72.1)	2604 (65.8)	0.112
**Ground-glass-opacity**	109 (82.6)	2757 (68.8)	0.001*	111 (75.5)	2710 (68.5)	0.071
**Centrilobular nodules with tree-in-bud**	87 (65.9)	1397 (34.9)	<0.001*	94 (63.9)	1344 (34.0)	<0.001*
**Paratrachealadenopathy**	66 (50.0)	1459 (36.4)	0.001*	61 (41.5)	1437 (36.3)	0.199
**Fibrosis**	22 (16.7)	1202 (30.0)	0.001*	34 (23.1)	1196 (30.2)	0.065
**Parenchymal Calcification**	12 (9.1)	754 (18.8)	0.005*	24 (16.3)	750 (18.9)	0.425

G1, patients with culture-positive PTB in derivation group; G2, patients other pulmonary diseases in the derivation group; G3, patients with culture-positive PTB in validation group; G4, patients other pulmonary diseases in the validation group.

Data are expressed as number (%).

s1, apical segment; s2, posterior segment right upper lobe; s1+s2, apico-posterior segment left upper lobe; s3, anterior segment of right upper lobe or left upper lobe; s4, lateral segment of right middle lobe or superior segment of left lingual lobe; s5, medial segment of right middle lobe or inferior segment of left lingual lobe; s6, superior segment of right or left lower lobe; s7, medical basal segment of right lower lobe; s8, anterior basal segment of right lower lobe; s7+8, medial-anterior basal segment of left lower lobe; s9, lateral basal segment of right or left lower lobe; s10, posterior basal segment of right or left lower lobe.

*Indicates statistical significance between G1 and G2 in derivation phase or between G3 and G4 in validation phase, *P*<0.05.

Multivariate logistic regression identified multiple independent predictors of culture-positive PTB in the derivation group (Table 3). We then developed a “relative score”, which was based on the ratio of each estimated β to the lowest one (i.e., the estimated cavitation of s1, s2, s1+s2 of 5.060). The relative score was 2 for ratios >1 and <1.5, and 3 for ratios >1.5 and <2.5. If the β effect was inverse, the score assigned was negative. The relative score was used in the multivariate logistic regression model. The ROC curve derived from the multivariate logistic regression had an AUC of 0.997 (95% CI 0.991 to 1.000, *P*<0.001; Fig. 2). The best observed sensitivity and specificity was found at a cutoff score of 1. The model had a predictive ability with a sensitivity, specificity, positive predictive value, and negative predictive value for the derivation vs. validation phase of 98.5% vs. 99.3%, 99.7% vs. 99.9%, 92.2% vs. 98.6%, and 99.9% vs. 99.9% s, respectively (Table 4). Details of the scoring system for predicting culture-positive PTB (total score>1) and other pulmonary diseases (total score≤1) are shown in Table S1 in File S1. The predictive model combined the results of cavitation (positive in s1, s2, s1+s2), consolidation (positive in s1, s2, s1+s2), consolidation (positive in s6), consolidation (positive in s7, s8, s7+8, s9, s10), and cluster nodules/mass (positive in s1, s2, s1+2), and the relative scores were derived thereafter. The frequency of patients with culture-positive PTB based on the scoring system in the derivation and validation phases is shown in Table S2 in File S1.

**Table 3:** Multivariate logistic regression analysis in derivation phase (N = 4,140).

	Estimated β (Std. Err.)	Estimated Odds Ratio (95% CI)	*P*	Relative Score^a^
Cavitation s1, s2, s1+s2	5.060 (1.434)	157.6 (9.5, 2619.1)	<0.001*	1
Consolidation s1, s2, s1+s2	5.944 (1.487)	381.3 (20.7, 7037.9)	<0.001*	2
Consolidation s7, s8, s7+s8 s9, s10	−7.588 (1.529)	0.001 (0, 0.010)	<0.001*	−3
Clusters nodules/mass s1, s2, s1+s2	9.669 (1.428)	15826.1 (964.1, 259786.2)	<0.001*	3
Consolidation s6	11.728 (1.777)	123962.7 (3810.4, 4032857.2)	<0.001*	3

s1, apical segment; s2, posterior segment right upper lobe; s1+s2, apico-posterior segment left upper lobe; s6, superior segment of right or left lower lobe; s7, medical basal segment of right lower lobe; s8, anterior basal segment of right lower lobe; s7+8, medial-anterior basal segment of left lower lobe; s9, lateral basal segment of right or left basal lower lobe; and s10, posterior basal segment of right or left lower lobe.

a Relative score is based on the ratio of each estimated β with the lowest one (5.060) as base = 1.

The relative score was set as 2 when the ratio (β/5.060) was >1 and <1.5, and as 3 when the ratio was ≥1.5 and <2.5. Since the effect of consolidation of s7, s8, s7+s8, s9, s10 was inverse, the relative score was set as negative.

*Indicates statistical significance, *P*<0.05.

**Table 4:** Predictive ability of HRCT in derivation phase and validation phase.

	Derivation phase		Validation phase	
	Culture-positive PTB (n = 132)	Other pulmonary diseases (n = 4,008)	Culture-positive PTB (n = 147)	Other pulmonary diseases (n = 3,958)
**Predictive results from HRCT model** *				
Predicted culture-positive PTB	130	11	146	2
Predicted absence of PTB	2	3997	1	3956
**Predictive terms**				
Sensitivity	130/132 (98.5%)		146/147 (99.3%)	
Specificity	3997/4008 (99.7%)		3956/3958 (99.9%)	
False negative rate^a^	2/132 (1.5%)		1/147 (0.7%)	
False positive rate^b^	11/4008 (0.3%)		2/3958 (0.1%)	
Positive predictive value	130/141 (92.2%)		146/148 (98.6%)	
Negative predictive value	3997/3999 (99.9%)		3956/3957 (99.9%)	

*The cutoff value from the predictive score to classify patients as culture-positive PTB with total score>1 and other pulmonary diseases with total score≤1.

a False negative rate, 1-sensitivity;

b False positive rate, 1-specificity.

Examples of the scoring can be seen by referring to Table S1 in File S1. If the patient had negative cavitation s1, s2, s1+s2, consolidation s1, s2, s1+s2, consolidation s6, and cluster nodules/mass s1, s2, s1+2, but positive consolidation of s7, s8, s7+8, s9, s10, then the patient would receive a total score of −3 (0+0+(−3)+0+0). As another example, in this study a total score of 0 was based on the combination of the patterns in cavitation (positive in s1, s2, s1+s2), consolidation (positive in s1, s2, s1+s2), consolidation (positive in s6), consolidation (positive in s7, s8, s7+8, s9, s10), and cluster nodules/mass (positive in s1, s2, s1+2). Example combinations are (0, 0, 0, 0, 0), (1, 2, −3, 0, 0), (0, 0, −3, 3, 0), and (0, 0, −3, 0, 3). Thus, a patient with a total score of 0 (0+0+(−3)+0+3) may have negative cavitation s1, s2, s1+s2, consolidation s1, s2, s1+s2, and cluster nodules/mass s1, s2, s1+2, but positive consolidation s7, s8, s7+8, s9, s10 and consolidation s6.

Among the 47 patients in the validation phase who were smear negative, 34 patients received a total score of 3, nine received a total score of 2, three received a total score of 5, and one received a total score of 1 (Table S3 in File S1).

Multivariate logistic regression analysis in the subgroup of derivation phase for 633 out of 4140 patients with previous PTB (19 with culture-positive PTB and 614 with other pulmonary diseases), revealed that the relative score was similar to the total patients in the derivation phase (Table S4 in File S1).

Table 5 summarizes the post-test probability according to the given prevalence and predicted positive LR [5], [29]. In the derivation phase, the HRCT screening protocol identified that 3.2% patients had culture-positive PTB. The post-test probability was derived as 91.5% based on the predicted positive LR+ value of 328.33. In the validation phase, the HRCT screening protocol identified that 3.6% patients had culture-positive PTB, and the post-test probability was derived as 92.5%. Moreover, the post-test probability were also estimated as 94.5%, 91.0%, and 76.8% when the pre-diagnosed probability (or prevalence of culture-positive PTB) were high prevalence (5.0%), moderate prevalence (3%), and moderate-to-low prevalence prevalence (1.0%) (Table 5).

**Table 5:** Summary of post-test probability according to the prevalence and predicted positive likelihood ratio.

	Prevalence of culture-positive PTB	Prediction Score	Pre-test odds	LR+	Post-test odds	Post-test probability
Study population in derivation phase	3.2%^a^	1	0.033	328.33	10.82	91.5%
Study population in validation phase	3.6%^b^	1	0.037	328.33	12.26	92.5%
High prevalence	5.0%	1	0.053	328.33	17.28	94.5%
Moderate prevalence	3.0%	1	0.031	328.33	10.15	91.0%
Moderate-to-low prevalence	1.0%	1	0.010	328.33	3.32	76.8%

LR+, predicted positive likelihood ratio. The LR+ = 328.33 derived from the equation (sensitivity/1-specificity) with a sensitivity = 98.5% and specificity = 99.7% in derivation phase.

a The prevalence was calculated based on the culture-positive PTB probability in the derivation phase (132/4140).

b The prevalence was calculated based on the culture-positive PTB probability in validation phase (147/4105).

## Discussion

The rapid diagnosis of culture-positive PTB is critical for preventing spread of the disease. If CXR is the only means of diagnosis, the cost of isolation (over diagnosis) and nosocomial spread (under diagnosis) will be great. The use of GeneXpert for diagnosing PTB promises to provide rapid and accurate diagnosis, but the test cannot be performed without sputum [30]. Many patients in this study were not able to produce sputum in the ED, and while bronchoscopy can be used to obtain sputum it is invasive and also a source of nosocomial infection. Though HRCT is associated with the use of ionizing radiation the impact of this is minimal in most adult patients, and in this study patients younger than 18 were excluded in order to reduce the impact of radiation. Also, this study utilized spiral CT, and the radiation dose was approximately 7 mSV. Taking the cost of isolation rooms, training of personnel with the CT equipment, and training of the radiologist into consideration, HCRT is feasible and a more cost-effective method for allocating resources for the isolation of patients [10], [31]–[34].

Our model identified consolidation of s1, s2, s1+s2 and s6; cavitation of s1, s2, and s1+s2; and clusters of nodules in s1, s2, and s1+s2 as positive factors predictive of culture-positive PTB, while consolidation of s7, s8, s7+s8, s9, and s10 were negative factors. Together, these factors had an overall high sensitivity (130/132, 98.5%), specificity (3997/4008, 99.7%), high positive predictive value (130/141, 92.2%) and high negative predictive value (3997/3999, 99.9%). The high sensitivity and high specificity contribute to the OR being as high as 328.33 [13]. In addition, high post-test probabilities in high (94.5%), moderate (91.0%), and moderate-to-low (76.8%) prevalence areas were obtained.

The most important finding in this study is that non-cavitation such as consolidation in s1, s2, s1+s2, and s6 and clusters of nodules/mass in s1, s2, and s1+s2 were associated with the highest positive predictive score. These findings largely agree with recent HRCT studies showing that not only cavitation of s1, s2, and s1+s2 [8] but also consolidation in s1, s2, s1+s2, and s6 [25] and clusters of nodules/mass in s1, s2, and s1+s2 [8] are predictive of culture-positive PTB. This observation is also in accordance with other previous studies [35], [36]. Consolidation of s7, s8, s7+s8, s9, and s10 was a negative factor in our model. This is in accordance with the high frequency of bacterial pneumonia in the lower lobe (73.3%) as reported by Coelho et al. [37]. Meanwhile, Yeh et al. [8] reported that only 16% (13/84) of smear-positive and 15% (6/40) of smear-negative culture-positive PTB patients had consolidation in s7, s8, s7+s8, s9, or s10. Furthermore, CT findings of culture-positive PTB in immunocompromised patients, such as those with diabetes, are similar to our findings [38]. This supports that consolidation of s7, s8, s7+s8, s9, and s10 was also a negative factor in predicting culture-positive PTB in the prior study by Yeh et al. [8].

Ideally, a decision instrument would have 100% sensitivity, specificity, and negative predictive value, and no patients with the disease would be missed [39]. In our model, the sensitivity, specificity, and negative predictive value are all >95%. We utilized the given prevalence rates to test the ability of the model [5]. The high OR contributes to the high post-test probability in moderate and moderate-to-low prevalence areas.

As previously reported by Kanaya et al. [5], a post-test probability of 5% is the threshold for withholding empiric treatment for patients with suspected PTB but with negative sputum results. In contrast, in high to moderate prevalence areas the threshold is more conservative. In our study, high post-test probability was observed in moderate and moderate-to-low prevalence areas. This finding implies that our model may be useful in deciding to initiate treatment or isolation in patients with suspected culture-positive PTB in different prevalence areas if post-test probability is >60% in these areas. Conversely, in very low prevalence area the risk and cost benefit must be considered [14], [40].

Our HRCT predictive model also produced lower a false positive rate based on the results from the validation phase. This implies that the necessity of respiratory isolation could be better determined based on our HRCT screening protocol, thereby reducing unnecessary cost and manpower in the management of this specific population of patients in high to low prevalence areas.

### Limitations

Our study has several limitations. Its scope was limited to the reliability and reproducibility of the five variables predictive of culture-positive PTB, and we focused on adult patients in the ED. While the validation phase showed good results at our ED, the prediction model was not tested in other areas. Other areas such as those with a different prevalence of TB or AIDS may also have patients with different demographic and clinical characteristics to which this model may not be applicable. There were some differences in the range of scores between the derivation and validation groups. However, this difference didn't affect the results of the prediction model, and the sensitivity and specificity were high in the two phases implying that the results are reproducible in our hospital and in other regional hospitals. There are a number of potential sources of bias in this study. However, we have attempted to reduce the sources of bias in a number of ways [41]. 1) The observer (radiologist) was not aware of the culture results. 2) The observer was not aware of the clinical manifestations. 3) Discrepancies were resolved by consensus. 4) Multiple logistic regression analysis was used to obtain the β values. 5) The number of patients was large. 6) The results of the derivation phase are valid and generalizable to the target population (high post-test probability in the validation phase). 7) The kappa values for inter-observer and intra-observer reliability were >0.9, indicating excellent reliability. Finally, combined disease was a limitation of this study and needs further investigation.

## Conclusions

Our prediction model using HRCT, which is feasible to perform in the ED, can promptly diagnose culture-positive PTB in moderate and moderate-to-low prevalence areas.

## Supporting Information

File S1 **Supplementary tables (Tables S1–S4).**
(DOC)
