# Supplementary material for: A High Resolution Computer Tomography Scoring System to Predict Culture-Positive Pulmonary Tuberculosis in the Emergency Department
Source: PLoS One. 2014 Apr 11;9(4):e93847. doi: 10.1371/journal.pone.0093847 (PMC3984117; doi:10.1371/journal.pone.0093847)
Supplement: File S1 — Supplementary tables (Tables S1–S4). (DOC) [file pone.0093847.s001.doc]

**Table S1. Scoring system used in the derivation and** validation phases

|  | Combination of scores | | | | |
| --- | --- | --- | --- | --- | --- |
|  | Cavitation s1, s2, s1+s2 | Consolidation  s1, s2, s1+s2 | Consolidation s7, s8, s7+8, s9, s10 | Cluster nodules/mass s1, s2, s1+s2 | Consolidation s6 |
| Total score | (Score = 1) | (Score = 2) | (Score = -3) | (Score = 3) | (Score = 3) |
| -3 | 0 | 0 | -3 | 0 | 0 |
| -2 | 1 | 0 | -3 | 0 | 0 |
| -1 | 0 | 2 | -3 | 0 | 0 |
| 0 | 0 | 0 | 0 | 0 | 0 |
|  | 1 | 2 | -3 | 0 | 0 |
|  | 0 | 0 | -3 | 3 | 0 |
|  | 0 | 0 | -3 | 0 | 3 |
| 1 | 1 | 0 | 0 | 0 | 0 |
|  | 1 | 0 | -3 | 3 | 0 |
|  | 1 | 0 | -3 | 0 | 3 |
| 2 | 0 | 2 | 0 | 0 | 0 |
|  | 0 | 2 | -3 | 3 | 0 |
|  | 0 | 2 | -3 | 0 | 3 |
| 3 | 1 | 2 | 0 | 0 | 0 |
|  | 1 | 2 | -3 | 3 | 0 |
|  | 1 | 2 | -3 | 0 | 3 |
|  | 0 | 0 | -3 | 3 | 3 |
|  | 0 | 0 | 0 | 3 | 0 |
|  | 0 | 0 | 0 | 0 | 3 |
| 4 | 1 | 0 | 0 | 3 | 0 |
|  | 1 | 0 | 0 | 0 | 3 |
| 5 | 0 | 2 | 0 | 3 | 0 |
|  | 0 | 2 | 0 | 0 | 3 |
| 6 | 1 | 2 | 0 | 3 | 0 |
|  | 1 | 2 | 0 | 0 | 3 |
|  | 0 | 0 | 0 | 3 | 3 |
| 7 | 1 | 0 | 0 | 3 | 3 |
| 8 | 0 | 2 | 0 | 3 | 3 |
| 9 | 1 | 2 | 0 | 3 | 3 |

**Table S2. The frequency of patients with culture-positive PTB based on the scoring system in the** derivation and validation phases

|  | Derivation (n=4140) | | |  | Validation (n=4105) | | |
| --- | --- | --- | --- | --- | --- | --- | --- |
| Total† score | Culture-positive PTB, smear-positive | Culture-positive PTB, smear-negative | Other pulmonary diseases |  | Culture-positive PTB, smear-positive | Culture-positive PTB, smear negative | Other pulmonary diseases |
| *N* | *108* | *24* | *4008* |  | *100* | *47* | *3958* |
| -3 | 0 | 0 | 991 |  | 0 | 0 | 963 |
| -2 | 0 | 0 | 0 |  | 0 | 0 | 0 |
| -1 | 0 | 0 | 300 |  | 0 | 0 | 315 |
| 0 | 1 | 0 | 2672 |  | 0 | 0 | 2668 |
| 1^‡^ | 1 | 0 | 34 |  | 0 | 1 | 10 |
| 2 | 1 | 0 | 5 |  | 6 | 9 | 2 |
| 3* | 7 | 17 | 6 |  | 6 | 34 | 0 |
| 4 | 6 | 0 | 0 |  | 5 | 0 | 0 |
| 5 | 22 | 4 | 0 |  | 19 | 3 | 0 |
| 6 | 40 | 3 | 0 |  | 38 | 0 | 0 |
| 7 | 0 | 0 | 0 |  | 0 | 0 | 0 |
| 8 | 1 | 0 | 0 |  | 1 | 0 | 0 |
| 9 | 29 | 0 | 0 |  | 25 | 0 | 0 |

The relative score from the HRCT model was set as 2 for consolidation in s1, s2, and s1+s2, 3 for consolidation in s6, -3 for consolidation in s7, s8, s7+8, s9, s10, 3 for clusters nodules in s1, s2, s1+2, and 1 for cavitation in s1, s2, and s1+2.

^†^ Total score was the summation by the relative score of consolidation of s1, s2, and s1+s2, consolidation of s6, consolidation of s7, s8, s7+s8, s9, and s10, clusters of nodules at s1, s2, s1+s2, and cavitation of s1, s2, and s1+s2.

^‡^ A total score > 1 was classified as culture-positive PTB, and a total score of ≤ 1 as other pulmonary diseases.

*Two patients with combined disease, culture-positive PTB with bacterial infection (n=1) and culture-positive PTB with lymphoma (n=1) were categorized as culture-positive PTB. The score of each patient was 3.

**Table S3. Total scores of the 47 smear-negative patients in the validation phase**

|  |  | Combination of scores | | | | |
| --- | --- | --- | --- | --- | --- | --- |
| Numbers of patients | Total score | Cavitation s2, s1, s1+2 | Consolidation  s1, s2, s1+s2 | Consolidation s7, s8, s7+s8, s9, s10 | Cluster nodules/mass s1,s2, s1+s2 | Consolidation s6 |
| 1 | 1 | 1 | 0 | -3 | 3 | 0 |
| 8 | 2 | 0 | 2 | 0 | 0 | 0 |
| 1 | 2 | 0 | 2 | -3 | 0 | 3 |
| 24 | 3 | 0 | 0 | 0 | 0 | 3 |
| 9 | 3 | 0 | 0 | 0 | 3 | 0 |
| 1 | 3 | 1 | 2 | 0 | 0 | 0 |
| 2 | 5 | 0 | 2 | 0 | 3 | 0 |
| 1 | 5 | 0 | 2 | 0 | 0 | 3 |

**Table S4. Multivariate logistic regression analysis in the subgroup of the derivation phase of 633 out of 4140 patients with previous PTB (19 with culture-positive PTB and 614 with other pulmonary diseases)**

|  | Estimated β  (Std. Err.) | Estimated Odds Ratio  (95% CI) | *P* | Relative Score^a^ |
| --- | --- | --- | --- | --- |
| Cavitation s1, s2, s1+s2 | 5.004 (1.459) | 245.61 (14.077, 4285.4) | < 0.001^*^ | 1 |
| Consolidation s1, s2, s1+s2 | 6.216(1.524) | 500.53 (25.258, 9918.8) | < 0.001^*^ | 2 |
| Consolidation s7, s8, s7+s8, s9, s10 | -7.644 (1.570) | 0 (0,0.100) | < 0.001^*^ | -3 |
| Clusters nodules/mass s1, s2, s1+s2 | 10.416 (1.562) | 33382 (1561.6, 713649) | < 0.001^*^ | 3 |
| Consolidation s6 | 12.002 (1.863) | 163042 (4227.9, 6287527) | <0.001^*^ | 3 |

s1, apical segment; s2, posterior segment right upper lobe; s1+s2, apico-posterior segment left upper lobe; s6, superior segment of right or left lower lobe; s7, medical basal segment of right lower lobe; s8, anterior basal segment of right lower lobe; s7+8, medial-anterior basal segment of left lower lobe; s9, lateral basal segment of right or left basal lower lobe; and s10, posterior basal segment of right or left lower lobe.

^a^Relative score is based on the ratio of each estimated β with the lowest one (5.004) as base = 1.

The relative score was set as 2 when the ratio (β/5.004) was > 1 and < 1.5, and as 3 when the ratio was ≥ 1.5 and < 2.5. Since the effect of consolidation of s7, s8, s7+s8, s9, s10 was inverse, the relative score was set as negative.

^*^Indicates statistical significance, *P* < 0.05.
